# Supplementary material for: SFRP2 Overexpression Induces an Osteoblast-like Phenotype in Prostate Cancer Cells
Source: Cells. 2022 Dec 16;11(24):4081. doi: 10.3390/cells11244081 (PMC9777425; doi:10.3390/cells11244081)
Supplement: Supplementary file 1 [file cells-11-04081-s001.zip › cells-2074352-Figure S1.pdf]

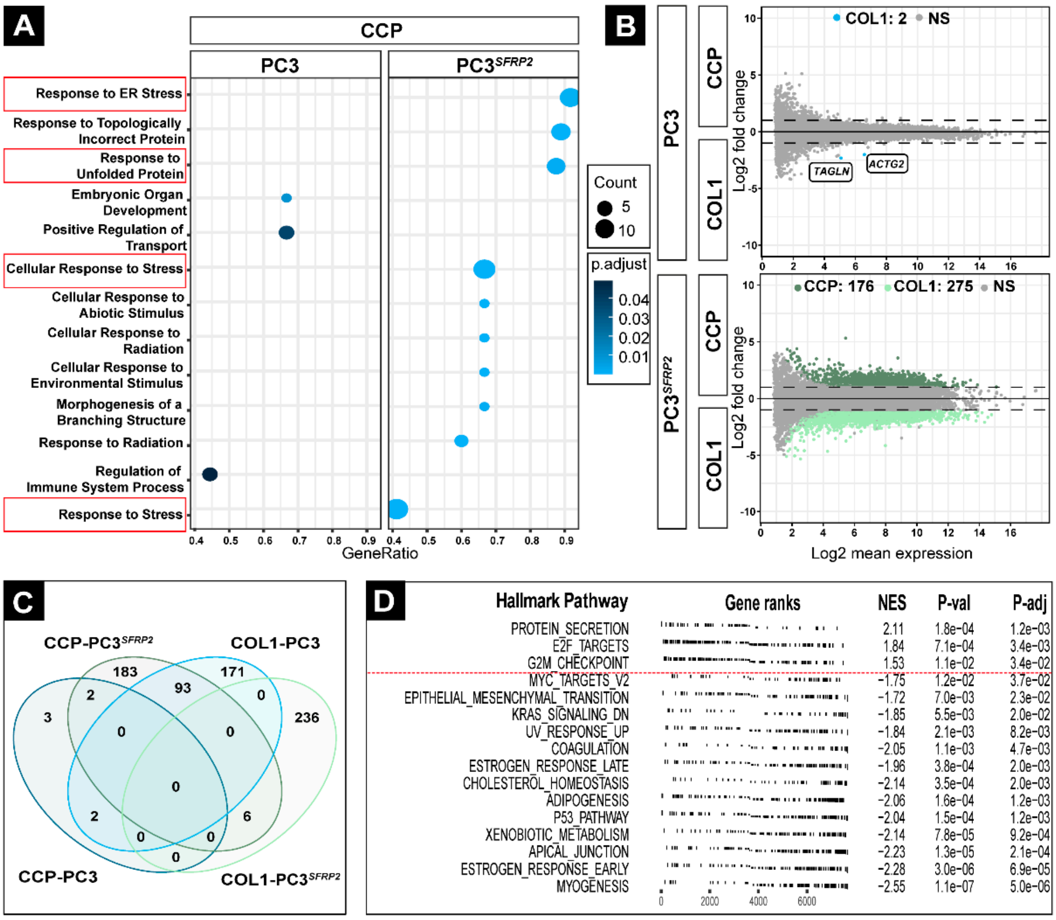


**Figure S1: Gene expression analysis results to understand the effect of CCP / COL1 on PC3 and PC3*SFRP2*.** GO analysis dot plot represents the effect of *SFRP2* overexpression on PC3 on CCP surface where it increases stress related pathways significantly **(A).** MA plots show that COL1 does not alter the transcriptome of control PC3 cells, but strongly affects the transcriptome of PC3*SFRP2* cells **(B)**. Venn diagram represents unique downregulated gene expression between all groups, with only cells seeded on a COL1-coated surface showing a marked difference **(C).** Hallmark gene sets show the comparison of CCP-PC3*SFRP2* **(NES > 1)** and COL1-PC3*SFRP2* **(NES < -1),** demonstrating the contribution of COL1 to EMT in SFRP2 overexpressing cells **(D).**
